# Anatomy into the battle of supporting or opposing reopening amid the COVID-19 pandemic on Twitter: A temporal and spatial analysis

**DOI:** 10.1371/journal.pone.0254359

**Published:** 2021-07-13

**Authors:** Lingyao Li, Abdolmajid Erfani, Yu Wang, Qingbin Cui

**Affiliations:** Department of Civil and Environmental Engineering, A. James Clark School of Engineering, University of Maryland, College Park, Maryland, United States of America; National University of Sciences and Technology (NUST), PAKISTAN

## Abstract

Reopening amid the COVID-19 pandemic has triggered a battle on social media. The supporters perceived that the lockdown policy could damage the economy and exacerbate social inequality. By contrast, the opponents believed it was necessary to contain the spread and ensure a safe environment for recovery. Anatomy into the battle is of importance to address public concerns, beliefs, and values, thereby enabling policymakers to determine the appropriate solutions to implement reopening policy. To this end, we investigated over 1.5 million related Twitter postings from April 17 to May 30, 2020. With the aid of natural language processing (NLP) techniques and machine learning classifiers, we classified each tweet into either a “supporting” or “opposing” class and then investigated the public perception from temporal and spatial perspectives. From the temporal dimension, we found that both political and scientific news that were extensively discussed on Twitter led to the perception of opposing reopening. Further, being the first mover with full reopen adversely affected the public reaction to reopening policy, while being the follower or late mover resulted in positive responses. From the spatial dimension, the correlation and regression analyses suggest that the state-level perception was very likely to be associated with political affiliation and health value.

## Introduction

A novel SARS-CoV-2 virus (COVID-19) that emerged in December 2019 has spread worldwide and become a pandemic [1]. As of June 10, 2021, more than 33.4 million cases and 598,000 deaths were reported in the United States, and the number of new cases is still high [2]. As the stay-at-home orders took effect in early April 2020, thousands of workforces were shut down, sports events were canceled, and universities and schools moved online. Beginning in mid-April, news media reported that anti-lockdown protests erupted across U.S. states [3]. Debates surrounding the necessities of lockdown orders and the appropriate time to reopen the country have raged for a long time. People were concerned that a prolonged lockdown would damage the economy and exacerbate existing social inequality in education and workforce environment [4], while others perceived that a temporal lockdown policy was necessary for slowing down COVID-19 spreads as well as ensuring a safe economic recovery environment [5]. Understanding the public risk propensity and dissecting the rival perceptions on reopening is of significance for the policymakers to cope with the challenges of enacting reopening policies.

Policymakers follow various approaches to determine the appropriate time to implement reopening phases. Premature reopening may increase the risks of contracting the virus in communities [6], but individuals may choose to undertake the risks due to living pressures. Tracking online perceptions on the reopening policy can provide meaningful insights for policymakers to comprehend how the online public thinks and behaves amid the COVID-19 pandemic. As social media establishes timely channels for online users to communicate information, crowdsourcing through social media plays a significant role in recognizing public opinions [7]. The advantages of leveraging social media to investigate public perceptions are manifold. First, social media offers policymakers opportunities to probe into a wealth of data that reflect people’s emotions and behaviors during a pandemic [8]. Second, social media can help construct a useful instrument to identify emergent responses, and therefore, policymakers can tailor their policies to the public demands. Last, social media-based approach supports the facilitation of timely track on online public perceptions that may exceed most conventional survey methods.

Social media data from Twitter, Facebook, and other web platforms have provided a rich source of information within the scientific community to investigate public perceptions relative to the COVID-19 crisis [9–11]. One broad application focuses on the areas of information dissemination and public engagement [11–13]. These studies have revealed that social media can support effective communication channels for government agencies or influencers to communicate important messages to the public. A recent survey based on 645 Italian clinicians reported that 47% of respondents answered that information shared on social media had a consistent impact on their daily practice [14]. Another study conducted by Chen et al. (2020) demonstrated that the dialogic loop on social media could help facilitate engagement through government accounts during the COVID-19 pandemic [12].

Social media postings contain a great deal of textual information. Textual analysis of vocabulary, semantic structures, and other textual features (e.g., text sentiment) conveys information that can be leveraged to assist in attitude survey, behavior analysis, and mental health detection [15–18]. Iglesias-Sánchez et al. (2020) [19] selected the case of COVID-19 quarantine in Spain and tracked the emotion changes based on online postings. Their study implied that isolation measures could have a significant impact on residents’ emotions, particularly arouse a noticeable response of anger emotion. Xue et al. (2020) [20] analyzed Twitter data in the early stages of this crisis, and their sentiment analysis result revealed that fear for the unknown nature of COVID-19 was dominant on Twitter.

Prior studies relative to the utility of social media in understanding public opinions show temporal and spatial variations [21–24]. Through the investigation of the geography of Twitter topics in London, Lansley and Longley (2016) [23] found that topics and attitudes expressed through tweets varied substantially across places and were associated with the demographic and socio-economic characters of the users. Koylu et al. (2018) [21] investigated the online public discourse and sentiment across space and time towards an immigration policy implemented in 2017, and their study manifested that such policy highlighted important partisan division within U.S. states. The opinion variations on political topics can be attributed to the inferred characteristics of online users. At this point, previous studies have also illustrated that demographic and socio-economic factors could exert an influence on public awareness, such as age, area of residence, income, educational level, and party affiliation [25–27].

Since the outbreak of COVID-19, several studies have analyzed the impacts of government policies on the public [28–30]. For example, Wei et al. (2020) [30] applied the interaction strategies and the evolutionary game analysis of the actions taken by the government and the public. Their study demonstrated that emergency response adopted by the government in the early stages of the pandemic could effectively contain the spread. For reopening policy, Nguyen et al. (2020) [31] quantified the effect of state reopening policies on daily mobility, and they observed an increase in mobility patterns during the reopening phase. Kaufman et al. (2020) [32] applied an interrupted time series to compare the rate of growth in COVID-19 cases after reopening to growth prior to the reopening. Their results revealed that states should delay further reopening until mask mandates were fully implemented. However, with the reviewed studies, the impacts of reopening policies have not been thoroughly investigated with social media data.

Building on the existing body of knowledge relative to the temporal and spatial analysis for online public opinions, this study aims to explore the potential of social media data (Twitter postings) to investigate online perceptions on reopening policy and demonstrate how the nature of the opinions varies according to the temporal and spatial characteristics. From the perspective of temporal analysis, online opinions on supporting the policy could vary in the appearance of influential news and events and might be affected by the timing (the first mover, follower, or late mover) to reopen the economy. From the perspective of spatial analysis, online perceptions might display significant differences geographically and could be associated with demographic and socio-economic characteristics. This study conducts correlation and regression analyses to unfold the demographic factors that can help explain the discrepancy and the consistency of public perceptions across U.S. states. As discussed, the findings of this study provide meaningful insights to understand how the online public reacted to the policies and further support policymakers to appropriately implement reopening policies.

## Materials and methods

### Data preparation and model framework

Twitter supports abundant data sources that can be accessed for capturing information given any topics. We used Twitter Standard Search API with the search term “reopen” to scrape tweets from April 17 to May 30, 2020. Other search terms, such as “open up,” “shut down,” may also contain information that implies a user’s perception on reopening policies. However, these terms were often used in tweets like “*business were shut down*,” and “*the park will open up next week*,” which were not indicative of inclinations to support or oppose reopening policy. As a result, using these terms may bring a large amount of noise to the dataset. More importantly, search terms including “lockdown” and “shut down” are not neutral terms as they were often appeared in tweets describing negative emotions during the lockdown period. For example, the tweet “*I’m so tired of being in lockdown*” describes the boredom emotion but does not adequately illustrate the user’s inclination to support or oppose reopening policy. Last, using other terms to download the data may result in a large variance of the textual information (e.g., the common topics of “reopen” and “lockdown” could be very different), which makes the machine classification process hard to implement.

For these considerations, we decided to use “reopen” as the search term to download tweet data. Since almost all states were fully reopened after May 30 [33], we restricted the search time range to May 30, 2020. Twitter provides two types of geographical data. One is the geo-tagged location, which is available when a user decides to share the location at time of tweet. However, only a small portion (<0.1%) of tweets were associated with geo-tagged locations in the dataset. The other type is registration location based on a user’s profile. Given that the research goal is to investigate the online perceptions on U.S. reopening policy, we filtered out those records with registration locations not implying a U.S. location. This filtering process resulted in a dataset with a total number of 2,407,911 records, in which 760,646 are unique tweets given that retweets are of the same textual content.

Then, we selected the 5,000 most frequently occurring unique tweets and manually classified each of them into support reopen (class 1), oppose reopen (class -1), or unrelated (class 0). Although previous studies show that retweeting behavior is not random [34, 35], we selected these tweets rather than a random subset to build the training dataset based upon two considerations. First, these 5,000 tweets are most likely to be reflective of impactful twitter data on the dataset as they received most retweets. More importantly, reviewing these 5,000 tweets could ensure more accurate classifications on the whole dataset. It was worth noting that they comprise 49.7% (1,196,274) of the full dataset (retweets have the same textual content). That being said, once the trained model gets a high training accuracy, retweets of these most occurring tweets are very likely to be correctly identified. Therefore, this treatment could ensure a higher classification accuracy on the whole dataset. Last, the testing data were randomly selected from the dataset so that the performance of the model (trained based on these selected tweets) on the testing set objectively reflects the accuracy on the whole dataset. More details regarding the training and testing process are presented in the S1 Appendix.

In the process of human labeling, two members from the research team labeled the same tweet for the first step. Once a tweet received the same label from both team members, it was considered as the final label for this tweet. Otherwise, another team member came to label this same tweet. The class of this re-labeled tweet was determined following the majority of the three manual labels. Among these 5,000 selected samples, 1,630, 1,950, and 1,420 samples were classified into class 1, class -1, and class 0, respectively. Meanwhile, we followed the same process and labeled another 2,339 unique tweets (different from the 5,000 tweets) that were randomly selected from the dataset to build a testing dataset. As a result, 744, 1,060, and 535 tweets were manually labeled as class 1, class -1, and class 0. Examples of the labels of tweets are attached in Table 4 in S1 Appendix.

**Fig 1:**
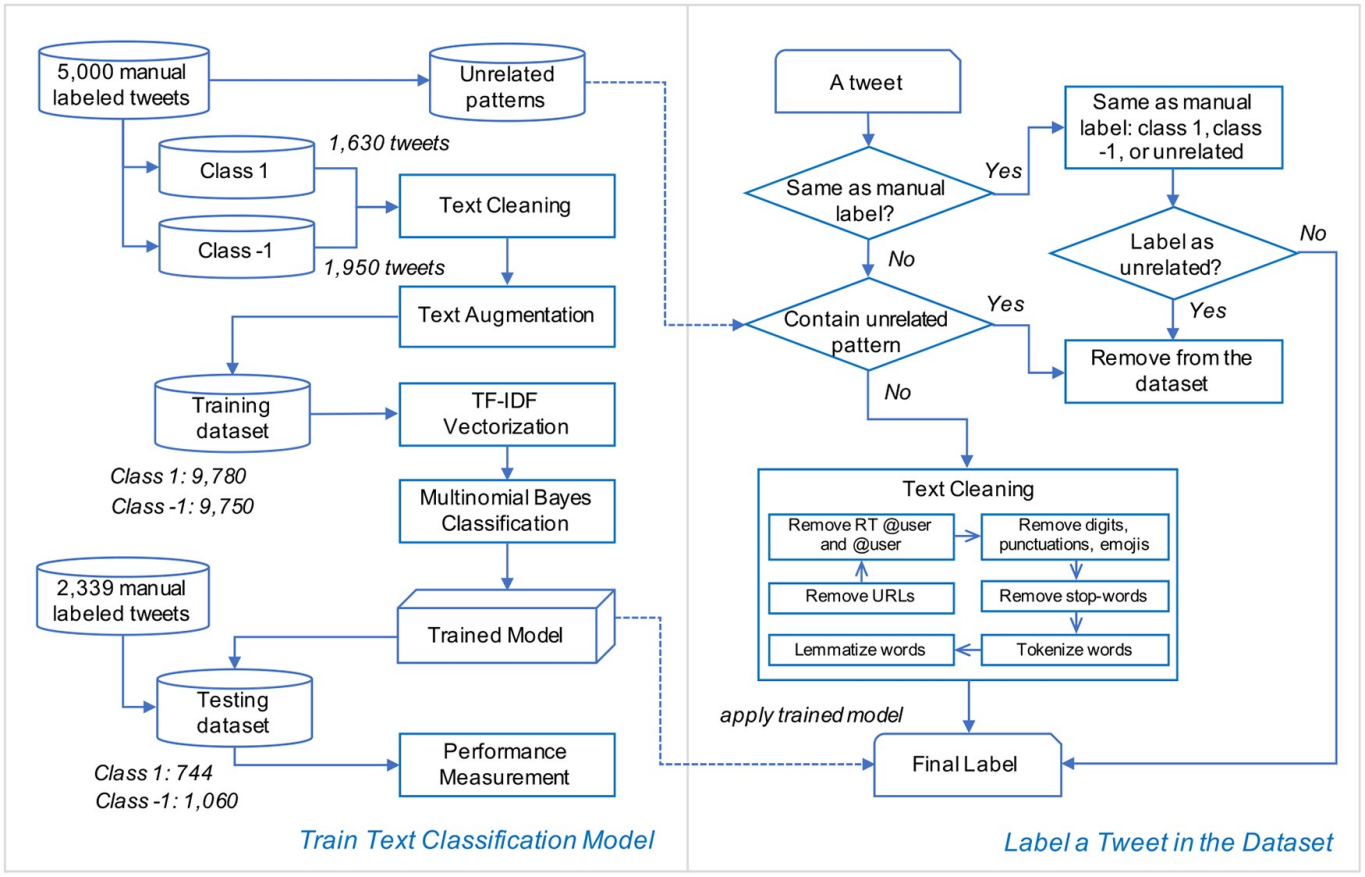
Model framework for the implementation of the proposed method.

As noted, “class 0” tweets contain the keyword “reopen” but do not imply the perception of supporting or opposing reopening policy. Examples include “national parks are set to reopen,” and “*Gyms and fitness centers can reopen on May 26 if they can meet safety protocols*.” In subsequent experiments, we found that a multi-class classification considering these tweets (class 0) largely reduced the testing accuracy from 73.0% to 58.3% possibly because they contain a large variance of textual information. Therefore, we determined to manually extract those textual patterns from 1,420 class 0 samples to remove tweets not informative of reopening perception. For example, we manually collected the word pattern “can reopen” from the tweet “Gyms and fitness centers can reopen on May 26 if they can meet safety protocols,” and used it to clear up “class 0” tweets from the dataset. Our manual collection of word patterns for filtering tweets is attached in the Section of Data Availability. As a result, the dataset was reduced from 2,407,911 to 1,591,216 with 450,450 unique tweets, even though a small portion of tweets in the dataset might not be fully cleaned. Correspondingly, we adjusted the training and testing datasets by filtering out those *“*class 0” samples. The model framework for the implementation of the proposed method is illustrated in Fig 1.

### Text cleaning and sample balance

Before text augmentation, we applied several steps to clean the tweets, as presented in the box “text cleaning” in Fig 1. We firstly removed short URLs, @username, RT @username, digits, emojis, and punctuations in a tweet. Then, we stripped those stop-words that were not informative, such as “the,” “is,” and “and.” Next, we tokenized each tweet into a list of separate words and characters. Since the words in a tweet can be written in different forms, we converted tokenized words to their base forms (also known as lemmatization). This cleaning process was completed with the aid of the Natural Language Toolkit (NLTK) python package [36].

As observed from Fig 1, samples labeled as class 1 and class -1 constitute 45.6% (1,632 out of 3,580) and 54.4% (1,948 out of 3,580) of the training dataset. Imbalance in the training dataset may result in a worse prediction performance for the minority class. Therefore, we utilized a simple text augmentation technique called Easy Data Augmentation (EDA) [37] to balance the distribution of class 1 and class -1 and increase the training data size. This text augmentation technique requires no NLP model to be pre-trained on any external dataset and is capable of improving the performance for a smaller dataset [37]. The EDA uses four operations 1) synonym replacement, 2) random insertion, 3) random swap, and 4) random deletion to increase the volume of labeled data [37]. Specific explanations and examples are presented in the S1 Appendix.

Following the recommendations by the study, we set the parameters for each of the four operations as α = 0.1, where α is a parameter that indicates the percentage of the words in a sentence that is changed [37]. However, the length of tweets can vary largely. Longer tweets have more words so that they can absorb more noise while maintaining original content. To compensate for this issue, as suggested by the research [37], the number of words changed in a tweet is defined as *n* = *αl*, where l is the length of a tweet. For shorter tweets, this EDA technique ensures that at least one word in the text is changed [37]. Further, we set the augmentation for class 1 and class -1 as 5 times and 4 times while conserved the original tweets. As a result, the data size for class 1 and class -1 was increased to 9,780 and 9,750, respectively, which were approximately equivalent in the training dataset.

### Text vectorization and classification

We applied the Term Frequency-Inverse Document Frequency (TF-IDF) and Word Embedding techniques to convert the tweets in the training dataset into vectors of features. TF-IDF is a popular term weighting method implemented in text similarity, text classification, and information retrieval [38]. Although TF-IDF cannot capture word positions or semantic meaning in a text, it is an efficient and useful algorithm to deal with a broad set of texts due to its simplicity and fast computation [39]. In TF-IDF, TF measures the number of words and their frequencies on each document, while IDF is incorporated to reduce the weights of common words in the corpus. The goal of using TF-IDF instead of the raw frequencies of words in a text is to scale down the impact of words that occur frequently and are hence empirically less informative. The representation of the TF-IDF method is given below [38]:

$$w(t,d)={tf\left(t,d\right)∙idf\left(t,D\right)=f}_{t,d}∙log(\frac{D}{d_{t}})$$ (1)

where *w*(*t*, *d*) represents the word *t*’s weight in tweet *d*, *f*_*t*,*d*_ denotes the frequency of word *t* in tweet *d*, *D* is the total number of tweets, and *d*_*t*_ is the number of tweets that word *t* appears.

Unlike TF-IDF method, Word Embedding techniques can help capture the semantic meanings of words in a context by converting each word into a pre-trained vector of features. They are often applied to compute text similarity or text classification. In this study, we adopted a popular Word Embedding technique called Word2Vec, which was released by the Google research team in 2013 [40]. The Word2Vec model was made up of a group of two-layer shallow neural networks and deployed with two architectures of continuous Bag-of-words and the Skip-gram to produce the vector representation for each word [40]. Word2Vec was trained using Google news, and each word vector has 300 dimensions [40].

As each tweet was converted to a vector of features, we applied several classifiers provided by scikit-learn python library to build the pipeline for text classification, including Bernoulli Naïve Bayes (BNB), Support Vector Machine (SVM) with Stochastic Gradient Descent (SGD), and Logistic Regression (LR) [41]. Since Multinomial Naïve Bayes (MNB) achieved the highest testing accuracy (Table 1), we specifically explained this algorithm in this section. MNB is a specialized Bayesian method assuming the data are multinomially distributed [42]. The distribution is parametrized by *θ*_*y*_ = (*θ*_*y*1_, *θ*_*y*2_, …, *θ*_*yn*_) for each class *y*, where *y* ∈ {−1, 1}, and the vectors of features can be obtained based on TF-IDF. *y* = 1 denotes that the tweet implies a perception of supporting reopen, while *y* = −1 denotes a perception of opposing reopen. *n* is the size of vocabulary (based on the number of words appeared in the tweets dataset). *θ*_*yi*_ = *P*(*x*_*i*_|*y*), i.e., the probability of word *x*_*i*_ appearing in a tweet given that the tweet belonging to class *y*. Bayes theorem defines the following relation given the class *y* and word *x*_1_ through word *x*_*n*_ [43]:

$$P\left(y|x_{1},x_{2},\ldots x_{n}\right)=\frac{P\left(y\right)P\left(x_{1},x_{2},\ldots x_{n}|y\right)}{P(x_{1},x_{2},\ldots x_{n})}$$ (2)

**Table 1:** Performance of different classifiers on the testing samples.

	MNB +	BNB +	SVM +	LR +	BNB +	SVM +	LR +
	TF-IDF	TF-IDF	TF-IDF	TF-IDF	Word2Vec	Word2Vec	Word2Vec
Precision							
Class 1	0.80	0.80	0.80	0.75	0.71	0.78	0.76
Class -1	0.65	0.63	0.64	0.60	0.53	0.59	0.58
Recall							
Class 1	0.72	0.69	0.70	0.68	0.59	0.65	0.63
Class -1	0.75	0.76	0.75	0.69	0.65	0.73	0.72
F1-score							
Class 1	0.76	0.74	0.75	0.71	0.65	0.71	0.69
Class -1	0.70	0.69	0.69	0.64	0.59	0.66	0.64
Training	93.9%	93.7%	93.5%	99.9%	68.6%	77.0%	78.0%
Testing	73.0%	72.0%	72.1%	68.1%	61.9%	68.2%	66.8%

The naive conditional independence makes an assumption that [43]:

$$P\left(x_{i}|{y,x}_{1},{\ldots x}_{i-1},x_{i+1}\ldots x_{n}\right)=P\left(x_{i}|y\right)$$ (3)

for all *i*, formula (3) can be simplified to [43]:

$$P\left(y|x_{1},x_{2},\ldots x_{n}\right)=p\left(y\right)\prod_{i=1}^{n}P\left(x_{i}|y\right)/P(x_{1},x_{2},\ldots x_{n})$$ (4)

As *P*(*x*_1_, *x*_2_, …*x*_*n*_) is a constant given the inputs, the estimation for *P*(*y|x*_1_, *x*_2_, …*x*_*n*_) can be denoted as [43]:

$$P\left(y|x_{1},x_{2},\ldots x_{n}\right)\propto P\left(y\right)\prod_{i=1}^{n}P\left(x_{i}|y\right)$$ (5)

$$\hat{y}={argmax}_{y}\;P\left(y\right)\prod_{i=1}^{n}P\left(x_{i}|y\right)$$ (6)

In this study, *P*(*y*) was the relative frequency of class 1 and class -1 in the training dataset, and *P*(*x*_*i*_|*y*) was estimated using TF-IDF technique.

Before applying the classifiers to opinion detection, we considered sentiment analysis given that sentiment techniques can classify the text into one of positive, negative, and neutral emotional categories. However, a prior study illustrated the differences between sentiment identification and opinion detection [44]. In this study, we applied a sentiment tool (TextBlob python package [45]) over the testing dataset and found that more than 60% of the classifications were not aligned with our manual labels. In some cases, sentiment identification is in line with reopening perception. For example, some users posted positive feelings when the restaurants reopened. This positive emotion also implies that the user supported the reopening policy. However, in other cases, sentiment identification may contradict with the opinion detection. For example, many users expressed that they were unhappy about the lockdown extension. This negative sentiment suggests a supportive attitude towards the reopening policy. Similarly, the positive sentiment might imply that online users supported the stay-at-home order, while the negative sentiment might indicate a surge in cases caused by reopening protests, both of which represented an attitude of opposing reopening. In summary, sentiment classifications could not represent users’ opinions towards the reopening policy and therefore were not applied in this study. Specific tweet examples are presented in Table 5 in S1 Appendix.

### Performance measurement

Precision, Recall, and F1-score were applied to assess the classification performance. Precision measures the fraction of true positive cases over the retrieved cases that a model predicts, while recall is the fraction of true positive cases over all the relevant cases. F-measure applied in this research uses the Harmonic Mean, known as F1-score. F1-score is a rating of test accuracy, representing a combination of Recall and Precision [46]. The mathematical formulas for Precision, Recall, and F1-score are presented in formula 7, 8, and 9, respectively. Performance on the testing samples of these classification pipelines is exhibited in Table 1.


Precision=TruePositive/(TruePositive+FalsePositive) (7)

Recall=TruePositive/(TruePositive+FalseNegtive) (8)

$$F1\;score=\frac{2\;Recall\times Precision}{Recall+Precision}$$ (9)

Overall, models that were trained based on TF-IDF outperform the trained models based on Word2Vec, demonstrated by both higher training accuracy and testing accuracy. A possible explanation is that the Word2Vec was not pre-trained using COVID-19 related topics, and thus it might not be able to capture the semantic meanings of some words in the dataset. Moreover, we simply took the average word embedding from each word vector to represent the tweet, and thus the trained model might ignore the importance of key words in a tweet and result in information loss. As a result, models trained on word embeddings might not discriminate the distinctions between tweets in some context. Among the four classifiers that were built on TF-IDF vectors, MNB slightly outperforms BNB and SVM, demonstrated by a higher F1-score on both classes and a higher testing accuracy. Although LR classifier achieves the highest training accuracy, it overfits the model and yields the lowest testing accuracy. For these considerations, we selected TF-IDF + MNB to build the pipeline and applied it to the whole dataset. However, it is apparent that those more sophisticated word embeddings and classifiers could easily have been applied once their performance warranted their choice in other cases.

## Results

### Temporal analysis

#### Temporal results

**Fig 2:**
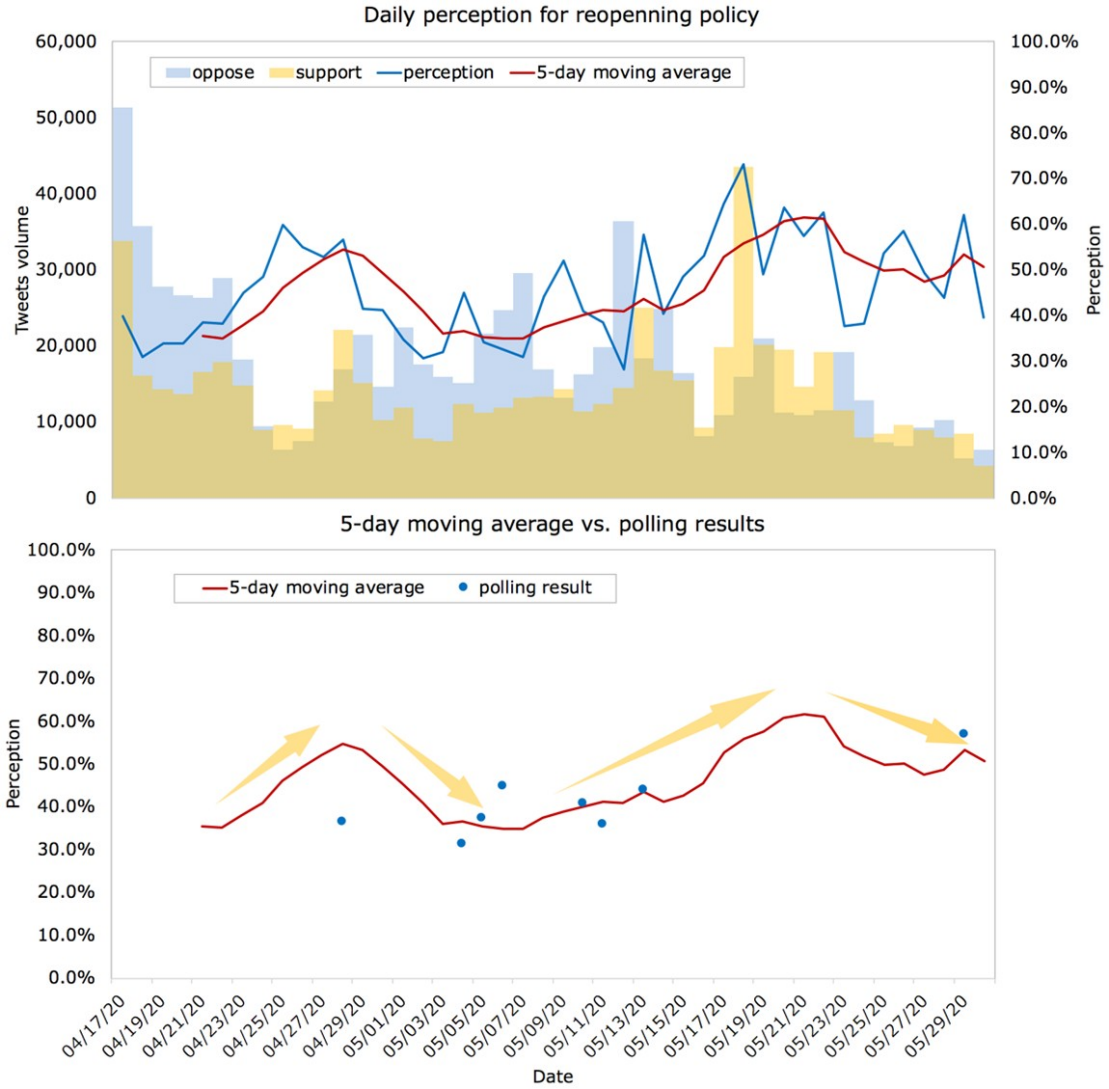
Daily perception and moving average. **a.** Daily perception and 5-day moving average for supporting reopening from April 17 to May 30, 2020. A perception < 0.5 indicates that more online users opposed reopening, while a perception > 0.5 implies that the majority supported reopening. The absolute volume of tweets indicating supporting reopening or opposing reopening is also presented. **b.** Comparison between the 5-day moving average perception with national polling results.

First, we computed the national-level daily perception based on the number of tweets supporting reopening divided by the total number of tweets each day. Fig 2 depicts the temporal changes in the study period. In particular, it presents a 5-day moving average to show a smoother trend of the perception changes. On most of the days, the perception was less than 0.5, demonstrated by a larger number of tweets implying opposing reopening (the blue bar is higher than the orange bar). In late April to early May, a larger proportion of online users perceived that it was too soon to reopen the country. However, when states such as Texas and Florida announced their reopening policies around April 25, tweets supporting reopening began to accumulate, and the volume exhibited a gradual increase. After May 25, when most states partially or fully reopened, the level of perception presented another increase. Overall, the perception after May 5 showed a more or less increase despite a short downturn from May 21 to May 25. This observation suggests that online users tended to switch to support reopening as the lockdown extended.

Analysis of Twitter data provide insights of online public’s responses to reopening policy. However, prior studies have shown that social media data might be an overrepresentation of young, educated, and urbanized population [47–49]. Specifically, Mislove et al. (2011) [47] raised a concern about whether Twitter could be representative of the overall population. Their research discovered that Twitter users significantly overrepresent the densely population regions. In subsequent studies, Barbera and Rivero (2015) [49] showed that Twitter users who discussed politics are likely to be male gender, to live in urban areas, and to have extreme ideological preferences. Mellon and Prosser (2017) [48] also suggested that Twitter and Facebook users are not representative of general population regarding political relevant discussions including vote choice, turnout, age, gender, and education.

Therefore, we compared the estimated perception with national polls to figure out how Twitter samples are biased in the representation of the public on reopening policy, as Fig 2 illustrated. We found that the estimated perception was different from national polls between May 5 to May 15. At other time during the study period, the results based on Twitter data were close to the national polls.

#### Popular news-driven tweets and their effects

News and events are of importance to drive public perceptions and often discussed in tweets. Numerous studies have showed that media coverage often exerts a significant impact on public perceptions by altering people’s exposure to information [50–52]. Inspired by these studies, we investigated how the important news especially political news and scientific news drove the discussion on Twitter and how they affected public perceptions on temporal horizon. We probed into the top 214 most retweeted tweets in the study period and extracted the news contents mentioned in the tweets. The top 214 tweets were retweeted 483,336 times in the study period (483,336 of 1,591,216, covering 30% data). Among these most retweeted 214 tweets, 152 are news-driven tweets, featured by a direct reference to a news event or the main text being followed by link to a news article.

In order to identify the popular news-driven tweets and their impacts on public perceptions, we first classified the 152 news-driven tweets into different categories. Specifically, we considered those tweet contents driven by news or reports mentioning political orders, plans, guidelines, statements, or announcements made by the president, governors, or other politicians as “political-news-driven.” In comparison, we considered those tweets containing scientific-related news or evidence, including scientific research findings, experimental data and reports, and guidance from experts, health officials, and research institutes as “scientific-news-driven.” For example, the following tweet is “scientific-news-driven” as it opens up a discussion based on the scientific evidence that the testing kits were not enough to guarantee a safe reopening environment.

*“We can’t safely reopen the economy until we can test millions of asymptomatic people and find out who can spread the virus*. *That requires a massive testing infrastructure and robust contact tracing we don’t yet have*. *The federal government must lead and stop blaming the states*. https://t.co/vYr3kAKMTz*”*

The following tweet reflects politics-related opinions that the administration shelved CDC guidance on how and when to reopen:

*“Reasons why CDC guidance was shelved*: *1*. *Guidelines say states should not reopen while their Covid cases are increasing*. *2*. *Trump admin wants states to reopen regardless*. *3*. *White House does not want to be accountable*, *and guidelines would make them so*. https://t.co/6VXqDaQSFc*”*

The human labeling process of the types of news was similar to the tweet labeling, as explained in Section 2.1 Data Preparation. A tweet was firstly labeled by two team members and checked by the third one if there was an inconsistency. As a result, 95 of 152 were identified as relative to political news, while 24 of them were relative to scientific news. It was also noted that some tweets could be driven by multiple types of news. Among the 152 tweets, five referred to both political news and scientific news. In addition to the tweets relative to political or scientific news, 38 of 152 were related to news that reported pandemic facts (e.g., death toll, new cases, testing), social events (e.g., protests), economic impact (e.g., unemployment). For example,

*“14*.*7% unemployment*. *It’s time to reopen America*. *We’re not going to be able to protect our elders or the sick if we have no economy*.*”*

*“Heads up re Alabama*…*’Alabama saw its largest single-day increase in new cases Monday*, *a little more than three weeks after the stay-at-home order expired on April 30 and two weeks after the state allowed restaurants and bars to reopen on May 11*.*’* https://t.co/TwBHTWXb0L*”*

**Fig 3:**
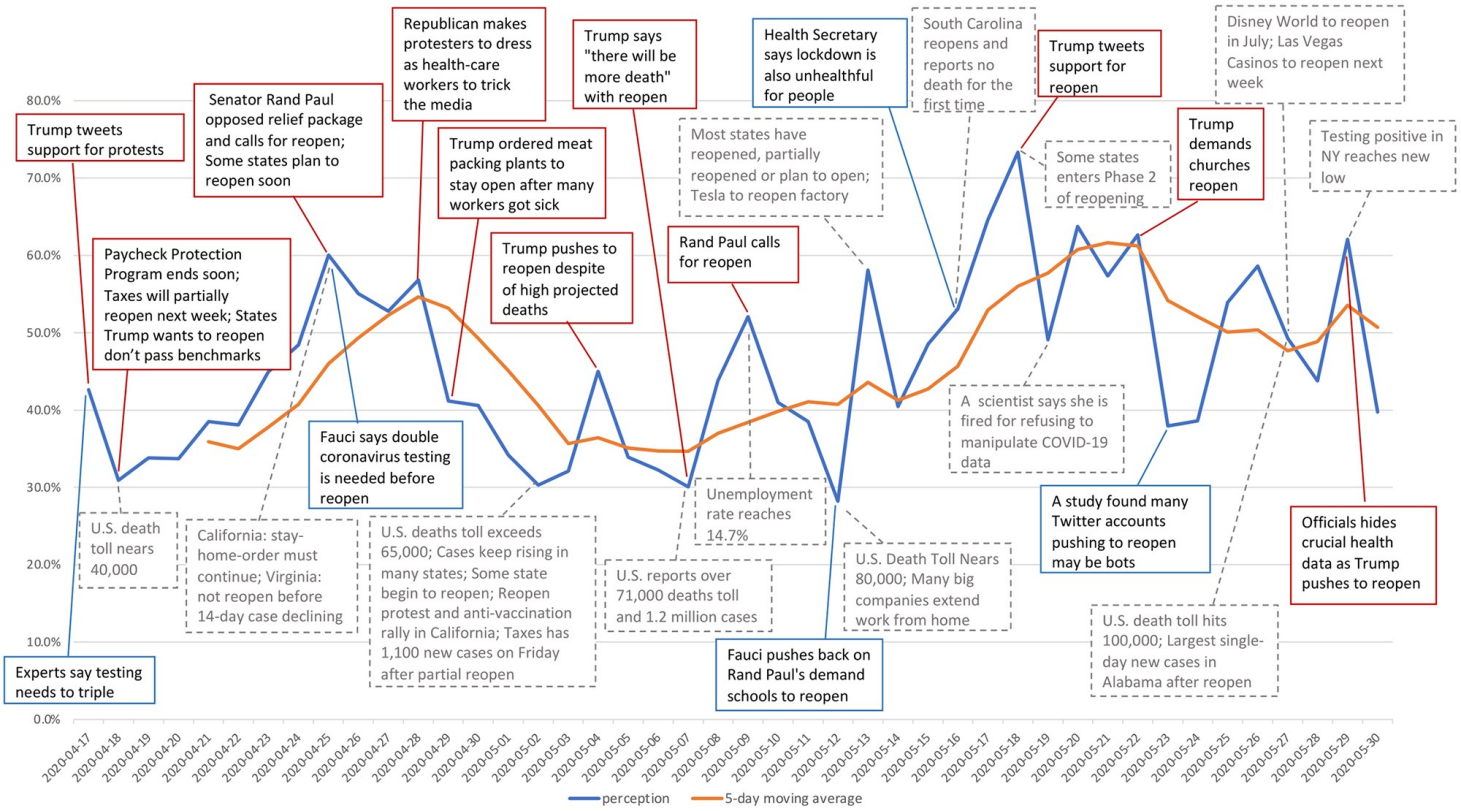
Daily perception and breaking news.

With the identifications of popular tweets, we summarized typical political (in red boxes), scientific (in blue boxes), and other types (in grey boxes) of news or evidence, as illustrated in Fig 3. We probed into the contents and the classifications (automatically classified by the trained model) of these popular tweets and found that news or opinions relative to reopen policy plans, announcements, economic recovery, and controlled outbreak often resulted in supporting reopening. However, news or opinions relative to pandemic outbreak, limited testing capacity, data manipulation, concerns for increasing cases and deaths, and safe recovery often led to a perception of opposing a premature reopening.

In addition, we noticed that 95 “political-news-driven” tweets were retweeted 175,908 times, while 24 “scientific-news-driven” tweets were retweeted a total of 56,573 times. Among the 95 “political-news-driven” tweets, 26 tweets (27.4%) with a total of 36,929 (21%) retweets express a positive sentiment of supporting reopening, while 69 tweets (72.6%) with a total of 138,979 (79%) retweets are negative about reopening. Among the 24 “scientific-news-driven” tweets, 3 tweets (12.5%) with a total of 3,108 (5.5%) retweets support reopening, and 21 tweets (87.5%) with a total of 53,465 (94.5%) retweets oppose reopening. This result manifests that the majority of both political news and scientific news that were extensively discussed on Twitter resulted in the view of opposing reopening on the temporal horizon. In particular, the scientific news implies an attitude of opposing reopening even more than the political news does, such as delivering alerts for premature reopen or highlighting data manipulation issues. As these tweets were widely recognized, it reflects that a substantial number of Twitter users acknowledged the same standpoint as the original tweet. Although this study did not cover all the tweets that contained political and scientific news, we recognized that investigation of these popular tweets could help support the analysis of how the political and scientific news drove people’s perception on reopening policy in the Twitter community.

#### Be the first mover, follower, or late mover

The appropriate time of reopening can be an additional driving factor on the temporal dimension that affected the perception. Being the first mover, follower or late mover is a significant question for policymakers to consider. A risk-based decision-making process should be taken into account to determine the appropriate time to reopen the economy [53]. This question has been examined in the literature of business strategy, and researchers raised the concern that being the first mover could result in potential hazards [54]. In this section, we attempted to evaluate the impacts on the public perception under multiple scenarios of reopening policies.

**Fig 4:**
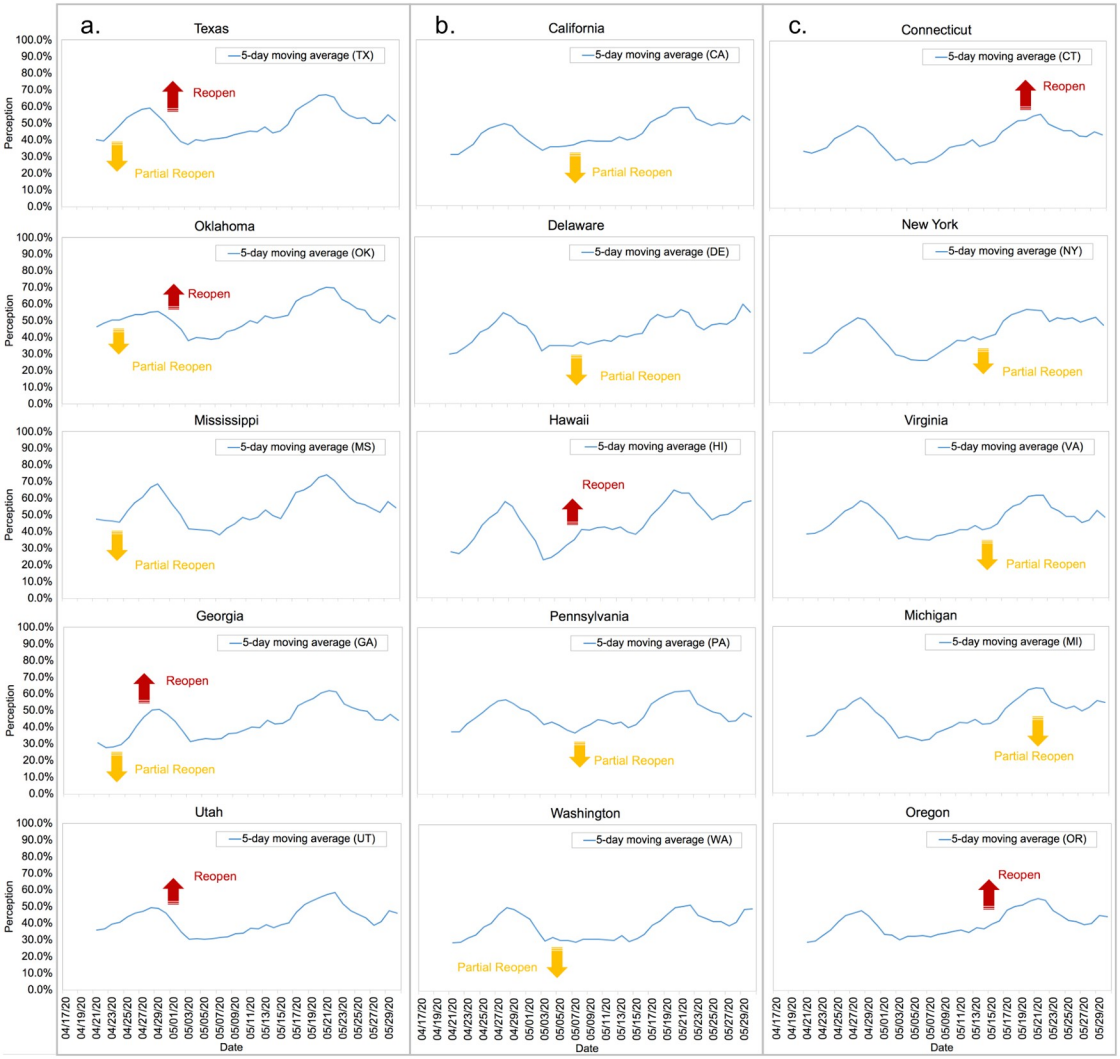
The impact of reopening policy on perception level. **a.** Group a: first mover (partially reopen or fully reopen before May 5). **b.** Group b: follower (partially reopen or fully reopen between May 5 and May 14). **c.** Group c: late mover (partially reopen or fully reopen after May 14). The upward arrow corresponds to the time of full reopen, and the downward arrow corresponds to the time of partial reopen.

We categorized the reopening policies into three groups based on the date when a reopening policy took effect, including “first mover” (before May 5), “follower” (from May 5 to May 14), and “late mover” (after May 14). Such division is mainly based upon the following two reasons. First, it generates three time periods with almost equal length. Second, we observed that May 5 and May 14 were the two time points that multiple U.S. states altered their reopen statuses [33]. Then we investigated the policy’s effects on the perception level, as exhibited in Table 2. Typical examples of dynamic perception changes are presented in Fig 4. The impact on perception was evaluated based on a 3-day average trend analysis after the implementation of a reopening policy, as presented below.

- *Negative = more than 3 percentage of negative reaction within 3 days*. *<-3%*
- *Slight negative = 1 to 3 percentage of negative reaction within 3 days*, *-1~3%*
- *Neutral = less than 1 percentage change in perception within 3 days*, *-1 ~1%*
- *Slight positive = 1 to 3 percentage of positive reaction within 3 days*, *1~3%*
- *Positive = more than 3 percentage of positive reaction within 3 days*, *>3%*

**Table 2:** Public reaction to reopening policy.

Group a:			Group b:			Group c:		
First Mover			Follower			Late Mover		
State	Partial	Full	State	Partial	Full	State	Partial	Full
TX	++	--	CA	/		CT		++
OK	++	--	DE	/		MI	++	
GA	++	--	RI	/		NY	++	
MS	++		KY	/		NM	++	
MT	+	-	AR	+	++	NH		++
TN	++	-	AZ	/	+	OR		++
UT		-	HI		++	VA		++
SC	++		PA	+		LA		++
MO		-	WA	+		FL		++
WV	++		VT	/	/	ID		++
NJ	--		NC	+		MA	++	
IL	--		SC		+	MD	++	
IN	--		WI		++	WY		++
OH	--		NH	++		KS		++
MN	--		FL	++		IN		--
KS	--		NV		--	WV		--
WI	--					OH		--
WY	--					NC		--
ND		+				KY		--
AL		++						
NE		++						

(Note: “++”: positive, “+”: slight positive, “/”: neutral, “-”: slight negative, “--”: negative.).

For the “first mover” group, the pattern manifests that, for 10 out of 21 states, the perception emerged on Twitter supported an early partial reopening policy (allowing some major sectors to reopen) but adversely reacted to a full early reopening policy (allowing every major sector to reopen). One possible explanation is that Twitter users were aware of the risks of increasing cases that might result from an early full reopening policy even though such policy aimed to reinvigorate a slumping economy. Moreover, 8 out of 21 states in the “first mover” group reacted negatively even to an early partial reopen, while only 3 out of 21 states (“AL,” “NE,” and “ND”) displayed a positive reaction to a fully early reopening policy. For the “follower” group, the observed pattern appeared to be consistent. A neutral or slightly positive reaction to reopening policies was reported from 16 out of 17 states in this group excepting “NV” state where an adverse reaction was observed. For the “late mover” group, the overall response was positive. 14 out of 19 states in this group showed a positive sentiment on the partial or full reopening policy. However, 5 states (“IN,” “WV,” “OH,” “NC,” and “KY”) displayed a negative reaction to a full reopening policy. In conclusion, the perception towards reopening policy exhibited a shift from negative to positive as the lockdown extended.

Overall, a partial reopening policy was likely to result in a more or less favorable increase on the perception level, possibly because people were concerned about the economic pressure under the COVID-19 pandemic. The result also suggests that in many U.S. states, the public willingness expressed on Twitter was not inclined to support a swift reopening strategy. By contrast, being the follower or late mover rather than the first mover of reopening policy was likely to be favored by Twitter users. However, the trends of different U.S. states could show variations as the COVID-19 outbreak hit with varying severity and time.

### Spatial analysis

#### Spatial results

**Fig 5:**
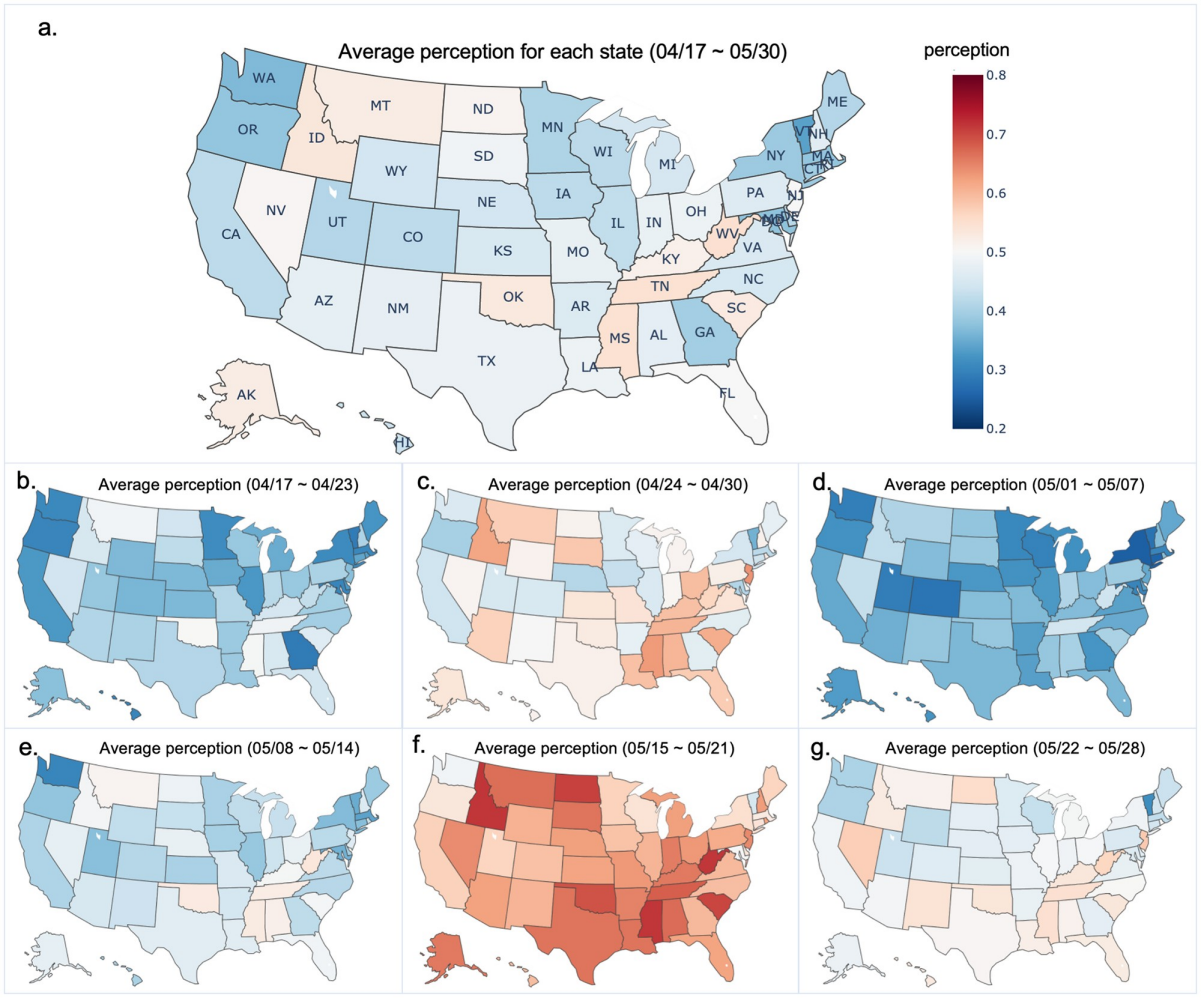
State-level average perception. **a.** Overall state-level average perception from April 17 to May 30, 2020. **b.** State-level average perception from April 17 to April 23. **c.** State-level average perception from April 24 to April 30. **d.** State-level average perception from May 1 to May 7. **e.** State-level average perception from May 8 to May 14. **f.** State-level average perception from May 15 to May 21. **g.** State-level average perception from May 22 to May 28. The figure was generated using the python choropleth graphing libraries (the code was released under MIT license) [55]. If the figure is similar, this figure is not identical to the original image and is therefore for illustrative purposes only.

From the spatial perspective, we focused on the analysis of state-level perception. We firstly binned the data and calculate the state-level perception based on the total number of tweets with users’ registration locations indicating the same state (e.g., “California, USA,” “Los Angeles,” “California,” and “Santa Monica CA” all indicate the California state). As shown in Fig 5, the average perception in the study period ranges from the lowest 33.8% to the highest 54.7% across the U.S. states. The five states with the highest perception for supporting reopening policy were West Virginia (WV, 54.7%), Missouri (MI, 54.6%), Tennessee (TN, 54.5%), Idaho (ID, 53.4%), and Oklahoma (OK, 53.1%). In comparison, the five states with the lowest perceptions were Vermont (VT, 33.8%), Washington (WA, 36.7%), Maryland (MD, 37.8%), Oregon (38.2%), and Massachusetts (38.2%). Overall, states located in the West, Midwest (especially East North Central), and Northeast region had a higher perception in comparison to states located in the South and Middle areas. Moreover, we presented the geographical distribution of state-level perception on a weekly basis in Fig 5. A continuous and consistent pattern observed from Fig 5 manifests that the majority of states located in the South and Midwest, especially West North Central held a higher perception to support reopening.

#### Correlation analysis

**Fig 6:**
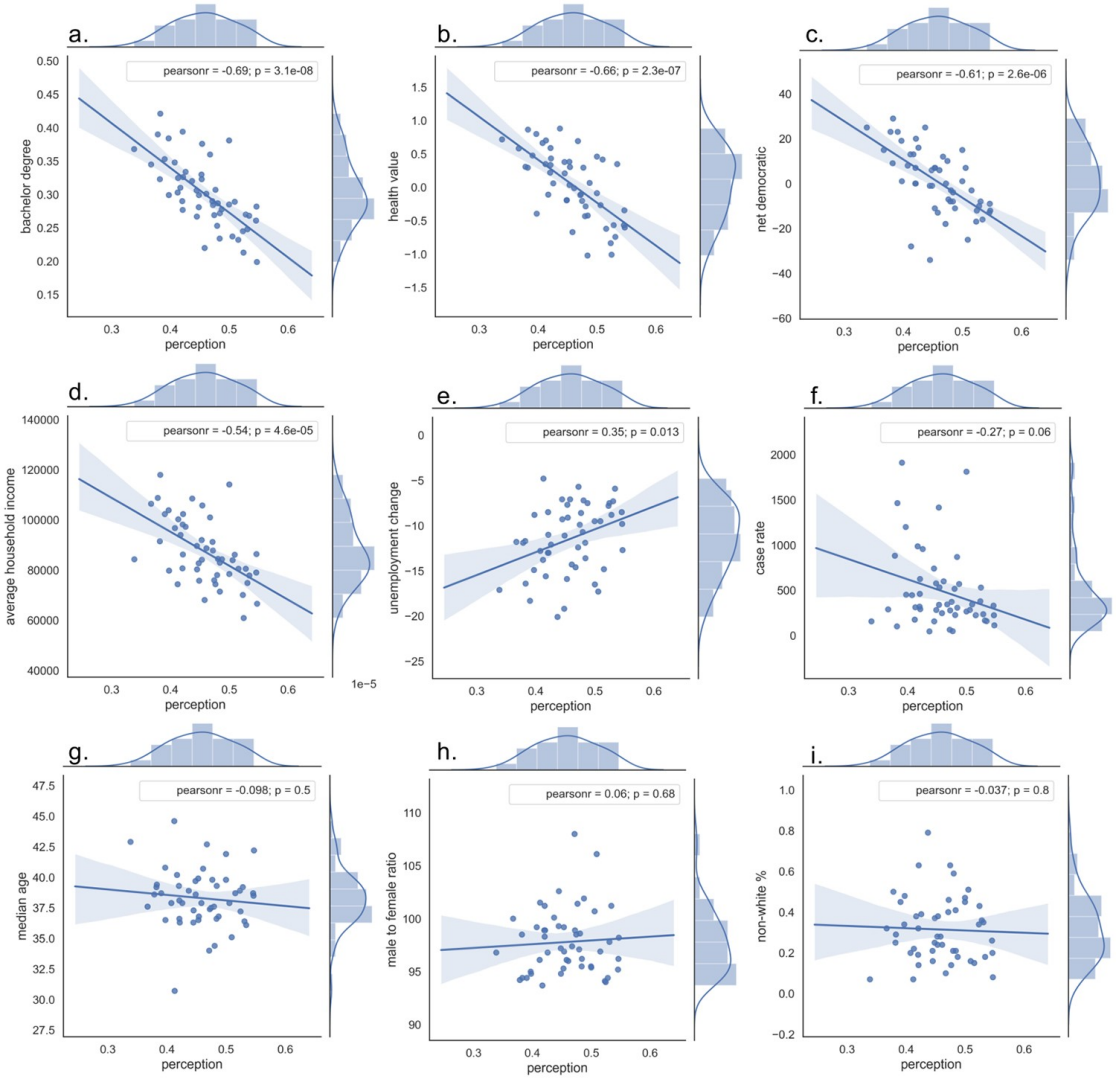
Correlation analysis with selected factors. **a.** Correlation with bachelor degree % (R = -0.69, p-value < 0.001). **b.** Correlation with health value (R = -0.66, p-value < 0.001). **c.** Correlation with net democratic (R = -0.61, p-value < 0.001). **d.** Correlation with average household income (R = -0.54, p-value < 0.001). **e.** Correlation with unemployment change rate (R = 0.35, p-value = 0.013). **f.** Correlation with case rate (R = -0.27, p-value = 0.060). **g.** Correlation with median age (R = -0.098, p-value = 0.497). **h.** Correlation with male to femal ratio (R = -0.06, p-value = 0.677). **i.** Correlation with non-white % (R = -0.037, p-value = 0.801).

We extended the spatial analysis to focus on the relations between the state-level perception and geodemographic attributes. This analysis aims to figure out geodemographic factors that were associated with the changes of the state-level perception. Previous studies revealed that socio-economic and political factors could affect the public perception, such as age, gender, race, income, educational level, party affiliation, and area of residence [25–27]. Therefore, we firstly performed a correlation analysis with nine selected geodemographic factors, including educational level (bachelor’s degree %) [56], health (health value 2018) [57], party affiliation (net democratic) [58], household income (average household income 2018) [59], age (median age 2018) [60], gender (male to female ratio 2018) [61], ethnic group (non-white percentage 2018) [62], and some factors related to the pandemic including the reported case rate (as of June 2) [63] and unemployment change (unemployment change from May 2019 to May 2020) [64]. The correlation results are exhibited in Fig 6.

According to Fig 6, the state-level perception exihibited a moderate and negative correlation with health value (R = -0.66, p-value < 0.001), bachelor degree (R = -0.69, p-value < 0.001), net democratic (R = -0.61. p-value < 0.001), and average household income (R = -0.54, p-value < 0.001). Twitter users in the states with higher health value, higher educational level, higher average household income, and more democratic inclined were less likely to support reopening policy. For those two selected factors directly referring to the COVID-19 pandemic, the state-level perception showed a weak correlation with unemployment change (R = 0.35, p-value = 0.013) and case rate (R = -0.27, p-value = 0.060). In a state with a higher case rate, Twitter users felt less inclined to support reopening policy. However, we observed that the perception appeared to be weakly and positively correlated with the unemployment rate change, indicating that users were likely to support reopening policy when the state had a lower unemployment rate. Among these investigated factors, the perception level didn’t show a significant correlation with median age, male to female ratio, and non-white ratio, demonstrated by p-value > 0.05. One interesting observation for Fig 6 was that the state-level perception showed a moderate correlation with those socioeconomic or political factors (education, party affiliation, health, income) within a state, weak correlation with the factors relative to the pandemic (unemployment change rate, case rate), and no significant correlation with demographic attributes (gender, age, ethnic group).

#### Regression analysis

Some selected geodemographic factors might be inter-correlated (e.g., bachelor degree, health value, household income, and net democratic). Therefore, we suspected that the selected attributes might not be statistically significant to estimate the perception level. Therefore, we performed a regression analysis to identify what identified socioeconomic and political factors (independent variables) could explain the changes of the perception level (dependent variable).

Ordinary Least Squares (OLS) was applied to fit a multi-linear regression model. The OLS model minimizes the sum of the squares of the differences between the calculated dependent variable (perception level) in the dataset and those predicted by the function. Since the OLS model assumes non-multicollinearity and homoscedasticity, we performed two diagnostic tests on the model, including the multicollinearity test and the heteroscedasticity test. Prior to feeding the data into the model, we selected the six features that showed moderate to strong correlations (explained in Section 3.2.2. Correlation analysis) with perception levels and applied the min-max scaling approach to normalize these input values into the range of (0, 1) to avoid that features in greater numeric ranges dominate those in smaller ranges.

The Variance Inflation Factor (VIF) quantifies the severity of multicollinearity. It provides an index that measures how much the variance (the square of the estimate’s standard deviation) of an estimated regression coefficient is inflated due to collinearity [65]. A VIF exists for each of the independent variable in a multiple regression model, and the VIF for i^th^ independent variable is represented as [65]:

$${VIF}_{i}=\frac{1}{1-R_{i}^{2}}$$ (10)

where $R_{i}^{2}$ is the R-square value obtained by regressing the i^th^ independent variable on the remaining independent. A VIF of 1 implies that there is no correlation between the i^th^ independent variable and the remaining variables, and thus the variance is not inflated. As a rule of thumb, VIF > 5 is caused for concern, and VIF > 10 indicates a serious collinearity problem [65]. In this study, we performed the VIF analysis on these six selected factors, and the VIF score for each factor is listed Table 3. The VIF score for the variable “bachelor degree %” is 7.297, which raises a concern about the colinearity issue for the regresssion model. Therefore, we removed this variable for subsequent regression analysis.

**Table 3:** Regression results.

Variable	VIF	Coef.	Std. Error	t	P > |t|	95% CI
bachelor’s degree	7.30					
health value	3.83	-0.514	0150	-3.43	0.001	[-0.817, -0.212]
net democratic	3.25	-0.527	0.173	-3.04	0.004	[-0.876, -0.178]
household income	4.45	0.159	0.206	0.77	0.443	[-0.256, 0.573]
unemployment rate	2.31	-0.113	0.140	-0.80	0.426	[-0.395, 0.170]
case rate	1.67	-0.027	0.135	-0.20	0.841	[-0.299, 0.244]

Meanwhile, the OLS model assumes that the observations have the same error variance. We performed a heteroscedastic analyiss using the White test [66]. Heteroscedasticity refers to the circumstance in which the conditional variance is not constant (Conditional variance is the variability of dependent variable for each value of the independent variables) [67]. According to the White test result, the F-statistic is 0.909, and the p-value is 0.581. This result does not reveal a significant goodness-of-fit and thus accepts the null hypothesis that the residuls are homoscedastic.

Based on the results of these two diagnostic tests, we selected the independent variables, including health value, net democratic, average household income, and unemployment rate, to perform the regression analysis. The number of observations is 50 (each U.S. state is considered as a data point in the model). As a result, the R-squared value is 0.55, implying that these identified independent variables can explain 55% of the dependent variable–perception level. The specific result of each independent variable is presented in Table 3.

According to Table 3, the t scores and p-values were used for the hypothesis testing of the coefficients–the variables of net democratic (p-value = 0.004) and health value (p-value = 0.001) have statistically significant p-value. It also means that these two variables were statistically significant in explaining the state-level perception.

From correlation and regression analyses, it is reasonable to conclude that the state-level perception was likely to be associated with the changes of party affiliation (net democratic) and health condition (health value), as these demographic characteristics within a state could affect its public perception of supporting reopening policy.

## Discussion

The COVID-19 pandemic has posted significant health threats to the U.S. society and weakened the domestic economy since its outbreak in March 2020 [68]. Reopening the country after the shutdown was a challenging decision for the policymakers to cope with. Premature reopening might trigger a second wave of widespread infections that could invalidate previous efforts [5], but a prolonged lockdown could dampen the economy and cause severe mental problems for people [69, 70].

The government’s decision to reopen the country should be subject to the inspection of public concerns, thoughts, and behaviors. Social media presents a rich source of information for the government agencies to detect the impact of their policies on the public. This study anatomizes the debate on Twitter surrounding the reopening policy from temporal and spatial perspectives. The goal of this study is to provide policymakers insights to understand the perception emerged on social media and its association with geodemographic factors.

In this study, we investigated more than 1.5 million tweets and employed NLP and machine learning techniques to classify the tweets into supporting or opposing reopening. With these classifications, we computed the perceptions and conducted the analysis from temporal and spatial dimensions. From the temporal dimension, our results show that popular political-news-driven and scientific-news-driven tweets could result in a view of opposing reopening. On top of that, we divided the reopening policies into three scenarios: first mover (before May 5), follower (May 5 ~ May 14), and late mover (after May 14). The result manifests that an early full reopening policy often exerted a negative influence on supporting reopening, but a late reopening policy or an early partial reopening policy could result in the positive sentiment on supporting reopening.

From the spatial dimension, we explored the correlations between the state-level perception and geodemographic factors. Our findings reveal a significant difference on the average state-level perceptions. The state-level perception showed a moderate negative correlation with socioeconomic and political factors, including education, health, party affiliation, and income. The state-level average perception also showed a weak correlation with factors relative to the COVID-19 pandemic, including the unemployment change rate and reported case rate. However, the perception was unlikely to be correlated with intrinsic demographic attributes on population, such as age, gender, and ethnic groups. More importantly, through the regression analysis, we found that the state-level perception was likely to be associated with the changes of party affiliation and health condition.

In this study, we demonstrate the feasibility of using social media data to track online public perceptions of reopening policy, and present a quantitative process to develop a pipeline to classify the tweets. This social media-based approach can be generalized to quantify the level of online perceptions on a policy or an event and has the advantages of rapidity, quantity, and spatial coverage. From practical perspectives, this study provides an instrument for the government agencies to detect the perception and insights on the public risk propensity, which further supports them to formulate a well-thought-out strategy.

Despite the aforementioned benefits, some limitations need to be highlighted. First, a small portion of unrelated tweets (class 0) might not be fully cleaned from the dataset since some textual patterns were not present in the collected samples. Second, using the key term “reopen” to download the data might result in some information loss. For example, this filtering would eliminate tweets, such as “*I don’t think the government should lift the stay-at-home order too soon*,” which expresses an opinion towards reopening but does not contain the key word. Third, since the testing accuracy is 73%, misclassifications could lead to biases in the result analysis. However, it was observed that the F1-scores of class 1 and class -1 were close, and therefore the impacts from misclassified tweets might be offset. Moreover, a large set of retweets were aligned with the same manual labels so the actual accuracy of labels could be much higher.

Ongoing and future work will first pay attention to the improvement of text classification models. One possible direction is to apply more sophisticated classifiers, such as deep neural networks. Another piece of future work will incorporate social media data from Facebook or Instagram into current findings and extend this study to establish a public perception tracking system, which may benefit government agencies, health officials, research institutes, and the residents.

## Conclusion

This study utilized a social media-based approach to investigate public perceptions towards reopening policy and anatomized the debate surrounding reopening policy on Twitter. This study investigated more than 2 million Twitter postings related to reopening policy in the date range from April 17 to May 30, 2020, and it built a pipeline for text classification using NLP and machine learning techniques. The result analysis was investigated from both temporal and spatial perspectives. From the temporal horizon, the results suggested that popular tweets mentioning political news and scientific news expressed more negative sentiment on supporting reopening. Moreover, being the first mover to reopen the state was more likely to result in a negative response to support reopening, while being the late mover triggered a more positive response. From the spatial horizon, the state-level perception exhibited a moderate and negative correlation with socioeconomic and political factors, including education level, health value, party affiliation, and household income. However, it did not show apparent correlations with intrinsic attributes of population like age, gender, or ethical group. The research findings provide the policymakers meaningful insights to track the public perception and understand how it reacts and interacts with related policies or news events and thus enable policymakers to enact appropriate solutions to implement reopening phases.

## Supporting information

S1 Appendix (DOCX)
