# Supplementary material for: Anatomy into the battle of supporting or opposing reopening amid the COVID-19 pandemic on Twitter: A temporal and spatial analysis
Source: PLoS One. 2021 Jul 13;16(7):e0254359. doi: 10.1371/journal.pone.0254359 (PMC8277023; doi:10.1371/journal.pone.0254359)
Supplement: S1 Appendix — (DOCX) [file pone.0254359.s001.docx]

**S1 Appendix**

**Examples of tweet labeling**

**Table 4. Examples of tweet labeling.**

| Tweet | Annot. 1 | Annot.  2 | Annot.  3 | Final Label |
| --- | --- | --- | --- | --- |
| REOPEN OUR COUNTRY! | 1 | 1 | / | 1 |
| South Carolina has reopened their state. And for the first time since March, they’ve reported NO COVID-19 deaths. RT if you think it’s time to REOPEN America! | 1 | 1 | / | 1 |
| How about we REOPEN, and whoever wants to say home can stay home! | 1 | 1 | / | 1 |
| There’s no way to safely reopen without massive testing. Yet we’re barely testing more people this week than last. And hospitals continue to report serious bottlenecks. If Trump ignores the experts and forces a premature reopening, Even more Americans will die. | -1 | -1 | / | -1 |
| So Texas has closed schools for the remainder of the year but want to reopen the club next week? WHAT😂😭 | -1 | -1 | / | -1 |
| The Trump admin is now projecting 3,000 daily deaths by June, and they are STILL pushing to “reopen.” We are now careening towards one 9/11-scale death event *per day.* This was all preventable w/ tests, tracing, & policy. Instead the GOP showered their friends w/ bailout money. | -1 | -1 | / | -1 |
| Our plan to safely reopen New York in phases will be guided by science and data. Read the full NY Forward plan here:  https://t.co/JEWlH0kyAa | 0 | 0 | / | 0 |
| BREAKING: BEACHES REOPEN - Jacksonville Mayor announces partial reopening of Duval County beaches and parks starting Friday at 5 p.m. https://t.co/HMLBI7tIMD | 0 | 0 | / | 0 |
| BREAKING NOW: AG Barr says it’s ‘time to start ROLLING BACK’ coronavirus restrictions 'in an orderly and sensible way' as more states plan to reopen.. DO YOU SUPPORT THE RE-OPENING OF THE COUNTRY? | 0 | 0 | / | 0 |
| I’m sure it’s just a total coincide that all the Reopen America bots and Republican bots and Trump bots and Russian bots all turned back on at the same time and are all in sync and pushing the same coordinated misinformation. A total coincidence. | 0 | -1 | -1 | -1 |
| Wow. Quite an exchange. GOP Sen Rand Paul suggested kids suffering less from coronavirus means schools can reopen. Dr. Fauci responded by saying, "I think we've got to be careful if we are not cavalier in thinking that children are completely immune to the deleterious effects." | 0 | -1 | -1 | -1 |

As described in the section of text classification, we investigated how the sentiment might help the analysis and ran a sentiment model (Textblob python library (45)) over the training and testing datasets. We found that more than 60% (1,127 tweets out of 1,804 testing tweets and 3,160 tweets out of 5,000 training tweets) of the classifications based on the sentiment analysis were not aligned with our manual labels. Following are a few examples showing that the sentiment results and opinions are different, as presented in Table 5.

**Table 5. Sentiment classification and manual labels.**

| Tweet | Sentiment score | Manual label |
| --- | --- | --- |
| Time to reopen the country | 0 (neutral) | 1 (support reopen) |
| Andrew Cuomo has enlisted the help of Bill Gates to reopen schools in New York and come up with a new way for distance education. There is nothing good that will come of this. At all. | 0.32 (positive) | -1 (oppose reopen) |
| Dear Donald Trump: Before you contradict Dr. Fauci in your rush to reopen schools, can you please get briefed by your own @CDCgov on how #Covid19 affects kids? My children and children across America should not be guinea pigs for a virus that we still don’t know much about. | 0.4 (positive) | -1 (oppose reopen) |
| Las Vegas mayor calls shut down "total insanity," demands Nevada governor reopen state now. | -0.1 (negative) | 1 (support reopen) |
| This guy is clinically insane. Illinois Governor Pritzker Threatens Business Owners with a Year in Prison if They Try to Reopen. | -1 (negative) | 1 (support reopen) |

**Training and testing process**

It is important to have a good setup of the training and testing datasets. In this study, we collected the 5,000 most frequently occurring tweets to build the training set and 2,339 randomly selected tweets to construct the testing set based upon the following reasons. First, the main purpose of training and testing is to achieve acceptable accuracy. Typically, researchers follow a 70-30% relation for training and testing. In our study, we followed this rule and selected 5,000 tweets and 2,339 testing to build the training set and the testing set, respectively. Second, our experiment showed that using 5,000 tweets could help achieve a comparatively high testing accuracy, and it is turning point that increasing the training size would not significantly improve the testing accuracy, as presented in Table 6. Third, these 5,000 selected samples received the most retweets and comprised 49.7% of the full dataset. Once the trained model achieved a high training accuracy, retweets of these most occurring tweets could be almost correctly classified. Last, we deployed a text augmentation technique that helps balance the training samples (as specifically described in the next appendix).

**Table 6. Training set size and accuracy.**

| Training dataset size | Training accuracy | Testing accuracy |
| --- | --- | --- |
| 1,000 | 95.1% | 67.4% |
| 2,000 | 89.9% | 69.4% |
| 3,000 | 87.8% | 70.1% |
| 4,000 | 88.2% | 72.0% |
| 5,000 | 88.2% | 72.0% |

**Text augmentation examples and accuracy improvement**

This study utilized a text augmentation technique called Easy Data Augmentation (EDA) (37) to balance the training data size. Operations and examples are listed in Table 7.

**Table 7. Operations and examples of EDA.**

| Operator | Ratio | Description | Example |
| --- | --- | --- | --- |
| Original tweet: *South Carolina has reopened their state. And for the first time since March, they’ve reported NO COVID-19 deaths. RT if you think it’s time to REOPEN America!*  Cleaned tweet: *south carolina reopen state first time since march theyve report no covid death rt think time reopen america* | | | |
| Synonym Replacement  (SR) | 0.1 | Randomly choose *n* words from the sentence. Replace each of these words with one of its synonyms. | South carolina reopen confederacy first time since march theyve report no covid death rt think time reopen america |
| Random Insertion  (RI) | 0.1 | Find a random synonym of a random word in the sentence. Insert that synonym into an arbitrary position in the sentence. Do this *n* times. | south carolina sentence reopen state first time since march theyve report no covid death rt think time reopen america |
| Random Swap  (RS) | 0.1 | Randomly choose two words in the sentence and swap their positions. Do this *n* times. | south reopen carolina state first time since march theyve report no covid death rt think time reopen america |
| Random Deletion  (RD) | 0.1 | For each word in the sentence, randomly remove it with the probability *p* | ~~south~~ carolina reopen state first time since march theyve report no covid death rt think time reopen america |

Table 8 exhibits the performance with the selected classifier (Multinomial Naïve Bayes) using the imbalance training dataset (3,580 tweets: 1,630 Class 1 tweets and 1,950 Class -1 tweets) and the balance training dataset improved by EDA technique (19,530 tweets: 9,780 Class 1 tweets and 9,750 Class -1 tweets). Results show that EDA helps improve the testing accuracy but also neutralizes the difference between classes based on F1-score and Recall improvements.

**Table 8. Performance on the testing dataset.**

|  |  |  | Precision | Recall | F1-score | Training | Testing |
| --- | --- | --- | --- | --- | --- | --- | --- |
| Imbalanced dataset | | |  |  |  |  |  |
| Class 1 | | | 0.73 | 0.83 | 0.78 | 88.2% | 72.0% |
| Class -1 | | | 0.70 | 0.56 | 0.62 |  |  |
| Balanced dataset | | |  |  |  |  |  |
| Class 1 | | | 0.80 | 0.72 | 0.76 | 93.9% | 73.0% |
| Class -1 | | | 0.65 | 0.75 | 0.70 |  |  |
